# Administering the maternal appeasing substance before slaughter to improve carcass characteristics of finishing cattle

**DOI:** 10.1093/tas/txae048

**Published:** 2024-04-02

**Authors:** Shea J Mackey, Reinaldo F Cooke, Autumn T Pickett

**Affiliations:** Department of Animal Science, Texas A&M University, College Station, TX 77845, USA; Department of Animal Science, Texas A&M University, College Station, TX 77845, USA; Department of Animal Science, Texas A&M University, College Station, TX 77845, USA

**Keywords:** bovine appeasing substance, carcass, feedlot cattle, stress

## Abstract

Two experiments evaluated carcass characteristics of finishing steers administered the maternal bovine appeasing substance (**mBAS**) prior to slaughter. In Exp. 1, 954 Angus-influenced finishing steers housed in 6 original pens were used. Each original pen was split into a pair of experimental pens 14.3 d ± 3 d prior to slaughter, in a manner that number of steers and average pen body weight (**BW**; 636 ± 4 kg) were similar. An oiler containing mBAS (Ferappease Finish Cattle 5%; FERA Diagnostics and Biologicals; College Station, TX) was added to one of the experimental pens 7 d prior to slaughter (*n* = 6), whereas the other pen did not contain an oiler (**CON**; *n* = 6). The oiler delivered 120 mL of mBAS/steer during a 7-d period. Steer BW was recorded 7 d prior to and during loading (final BW) to the packing plant. No treatment effects were detected (*P *≥ 0.51) for BW gain, final BW, and proportion of carcasses that graded Choice or Prime. Carcass dressing percentage was greater (*P *= 0.02) in mBAS compared with CON steers (65.9% vs. 64.2%; SEM = 0.5), which was not sufficient to impact hot carcass weight (**HCW**; *P *= 0.29). Incidence of dark-cutting carcasses did not differ between treatments (*P *= 0.23). In Exp. 2, 80 Angus-influenced finishing steers housed in 16 pens (5 steers/pen; 600 ± 4 kg of BW) were used. Pens were arranged in 4 rows of 4 pens/row, and rows were alternately assigned to receive an oiler containing mBAS (*n* = 8) or mineral oil (**CON+**; *n* = 8) 7 d prior to slaughter. Oilers were designed to deliver 120 mL/steer of mBAS or mineral oil during the 7-d period. Steer BW was recorded as in Exp. 1, and a blood sample was collected during exsanguination. No treatment effects were detected (*P *≥ 0.20) for BW parameters, carcass marbling score, backfat thickness, *Longissimus* muscle area, yield grade, and proportion of carcasses that graded Choice or Prime. Carcass dressing was greater (*P *= 0.02) in mBAS steers compared with CON + (60.6 vs. 59.6%; SEM = 0.3) but HCW did not differ (*P *= 0.47) between treatments. Plasma cortisol concentration was less (*P *< 0.01) in mBAS steers compared with CON + (11.7 vs. 20.8 ng/mL; SEM = 1.6). Incidence of dark-cutting carcasses did not differ (*P *= 0.53) between treatments. In summary, mBAS administration to finishing cattle using oilers during the last 7 d on feed alleviated the adrenocortical stress response elicited by the process of slaughter, which likely resulted in increased carcass dressing.

## Teaser

Feedlot cattle are exposed to several stressors during processing for slaughter that directly impact their carcass and meat quality traits. This study evaluated the maternal bovine appeasing substance, an analogue of the bovine appeasing pheromone, as a strategy to alleviate such stressors and improve carcass traits of feedlot cattle.

## Introduction

Feedlot cattle are exposed to several stressors during processing for slaughter that directly impact their carcass and meat quality traits (Kumar et al., 2023). Examples of such stressors include handling, transport, lairage, and exposure to new environments (Grandin, 1980), which elicit adrenocortical reactions that are catabolic and deplete muscle glycogen (Apple et al., 2005; Gregory, 2008). Stress has been associated with beef carcasses having higher pH, darker color, and less water holding capacity (Grandin, 1980; Cheng and Sun, 2008); therefore, management strategies to mitigate stress in feedlot cattle prior to and during slaughter are warranted.

The maternal bovine appeasing substance (**mBAS**) is a mixture of fatty acids that replicate the composition of the original bovine appeasing pheromone, and shown to alleviate the physiological consequences elicited by stressful management procedures (Cappellozza and Cooke, 2022). Research from our group reported that mBAS administration reduced cortisol concentrations and inflammatory reactions in beef calves upon weaning (Schubach et al., 2020) and in feedlot steers during the receiving period (Colombo et al., 2020). Cappellozza et al. (2020) also evaluated mBAS administration to *Bos indicus* bulls at the time of loading to the packing plant, and noted lower pH values in carcasses of bulls that received mBAS. These authors suggested mBAS as novel management to alleviate the stressors associated with slaughter, and that additional research was needed to characterize its benefits to carcass traits. Such studies include evaluation of *B. taurus* cattle representative of the US feedlot industry, and exposed to mBAS during the last week of feeding to mitigate the stress caused by handling cattle for truck loading (Scanga et al., 1998).

Administration of mBAS to cattle on feed should be passive and without physical handling or processing; otherwise, the stress elicited by these events will offset the benefits of mBAS. Oilers are commonly added to feedlot pens as self-treatment devices for insecticides (Barker et al., 2017), and can also be used to deliver mBAS to cattle prior to slaughter. Therefore, we hypothesized that mBAS administration using oilers will alleviate the stress caused by handling, transporting, and processing cattle for slaughter, resulting in improved carcass traits. Experiment 1 evaluated the use of oilers with mBAS on carcass characteristics of finishing cattle in a large-pen commercial feedyard. Experiment 2 investigated the use of oilers with or without mBAS in a small-pen research feedyard, and its effects on post-slaughter serum cortisol concentrations and carcass traits.

## Materials and Methods

Experiment 1 was conducted at a commercial feedyard (Pride Feeders; Adams, OK), and animals were cared for in accordance with acceptable practices as outlined in the Guide for the Care and Use of Agricultural Animals in Agricultural Research and Teaching (FASS, 2020). Experiment 2 was conducted at the Texas A&M—McGregor Research Center (McGregor, TX), and cattle were cared for in accordance with acceptable practices and experimental protocols reviewed and approved by the Texas A&M AgriLife Research, Agriculture Animal Care and Use Committee (#2023-006A).

### Experiment 1

A total of 954 Angus-influenced finishing steers housed in 6 original pens were assigned to this experiment. Steers were on a diet based on steam-flaked corn and implanted with trenbolone acetate + estradiol. Table 1 described the number of steers per pen, days on feed (**DOF**), body weight (**BW**), and days before slaughter when each original pen (120 × 40 m; drylot with no shade) was split into a pair of experimental pens for treatment administration. More specifically, steers within each original pen were weighed (636 ± 4 kg of BW; Table 1) and allocated to the experimental pens in a manner that number of steers and pen BW were similar. The pair of experimental pens had the same dimensions and general characteristics (90 × 20 m; drylot with no shade), and were not adjacent to each other to prevent physical contact of steers assigned to different treatments. An oiler (Prairie Phoenix Cattle Care System; Whitehorse, SD) containing mBAS (Ferappease Finish Cattle 5%; FERA Diagnostics and Biologicals; College Station, TX) was added to one of the experimental pens 7 d prior to slaughter, whereas the other pen did not contain an oiler (**CON**). The oiler was designed to deliver 120 mL of mBAS per steer during a 7-d period. Oilers were checked daily and mBAS was replenished according to dosage when necessary. This process was replicated across all 6 original pens within a 64-d period, resulting in 6 experimental pens receiving mBAS and 6 experimental pens serving as CON.

**Table 1.** Number of steers, days on feed (**DOF**), average body weight (**BW**), and days prior slaughter when 6 original pens were split into a pair of experimental pens and enrolled in Exp. 1^1^

Item	Head, *n*	DOF	BW, kg	Days prior to slaughter, d
Pen 1	144	189	662	13
Pen 2	208	260	608	16
Pen 3	147	206	627	23
Pen 4	189	169	627	14
Pen 5	128	214	648	9
Pen 6	138	226	647	11
Mean ± SE	159 ± 13	210 ± 13	636 ± 8	14.3 ± 2.0

^1^Steers within each original pen were weighed and allocated to a pair of experimental pens in a manner that number of steers and average pen BW were similar.

All steers from each original pen were slaughtered on the same day at a commercial plant (National Beef Packing Company; Liberal, KS). Steers from the CON experimental pen were weighed (final BW) and loaded into livestock trailers in the morning (up to 36 steers/trailer). Immediately after the CON experimental pen was loaded, steers from the mBAS experimental pen were assigned to the same process. Trailers traveled together for 48 km to the packing plant where the CON experimental pen was unloaded first. In the packing plant, the CON and the mBAS experimental pens were maintained in separate non-adjacent pens according to treatment, and slaughtered within 6 h after arrival. Hot carcass weight (**HCW**), carcass quality grading, and incidence of dark cutters were recorded by the packing plant (experimental pen basis), and HCW used to calculate carcass dressing according to the final BW of the respective experimental pen.

### Experiment 2

A total of 80 Angus-influenced finishing steers (600 ± 4 kg of BW) were assigned to this experiment. Steers were also implanted with trenbolone acetate + estradiol, and were housed in 16 pens (5 steers/pen; 50 × 10 m; drylot with no shade) receiving a diet based on rolled corn for 160 d. Pens were arranged in 4 rows of 4 pens/row, and rows were alternately assigned to receive an oiler (1 oiler/pen; Prairie Phoenix Cattle Care System) containing mBAS (Ferappease® Finish Cattle 5%) or mineral oil (placebo; **CON+**) 7 d prior to slaughter (n = 8 pens/treatment) as in Colombo et al. (2020). The oiler was designed to deliver 120 mL of mBAS or mineral oil per steer during a 7-d period, checked daily and replenished when necessary.

Steers BW was recorded 7 d prior to slaughter and at the time of loading (0700 h) to the packing plant (Tyson Foods; Amarillo, TX). As in Exp. 1, steers from CON + pens were loaded into livestock trailers (20 CON + steers/trailer) followed by mBAS pens (20 mBAS steers/trailer). All trailers traveled together for 720 km to the packing plant where CON + steers were unloaded first, whereas CON + and mBAS pens were maintained in separate non-adjacent pens. All steers were slaughtered within 6 h after arrival. Upon slaughter, a blood sample was collected during exsanguination into a blood collection tubes (Vacutainer, 10 mL; Becton Dickinson, Franklin Lakes, NJ) containing freeze-dried sodium heparin and hot carcass weight (**HCW**) was recorded. Blood samples were placed immediately on ice, centrifuged (2,500 × g for 30 min; 4 °C) and plasma stored at −80 °C on the same day of collection. Plasma samples were analyzed in duplicates for concentrations of cortisol (Salimetrics Expanded Range, High Sensitivity 1-E3002, State College, PA), with intra- and inter-assay CV of, respectively, 2.1% and 3.7%. After a 24-h chill, trained personnel assessed carcass characteristics including backfat thickness at the 12th-rib, marbling, and *Longissimus muscle* (**LM**) area.

### Statistical analyses

Data from both experiments were analyzed using a complete randomized design using pen as the experimental unit, and Satterthwaite approximation to determine the denominator degrees of freedom for tests of fixed effects. Quantitative data were analyzed using the MIXED procedure of SAS (SAS Inst. Inc., Cary, NC), whereas binary data (carcasses classified as dark cutters or that graded Choice or Prime) were analyzed using the GLIMMIX procedure of SAS (SAS Inst. Inc.) with a binomial distribution and logit link function. All model statements contained the fixed effect of treatment. Models from Exp. 1 included pen(treatment) as random variable, and models from Exp. 2 included pen(treatment) and steer(pen) as random variables. All results are reported as least square means. Significance was set at *P* ≤ 0.05 and tendencies were determined if *P* > 0.05 and ≤ 0.10.

## Results and Discussion

### Experiment 1

Initial BW did not differ between CON and mBAS as designed (*P *= 0.75), whereas BW gain and final BW were also similar (*P *= 0.51) between treatments (Table 2). Despite the benefits of mBAS to growth of weaned and feedlot receiving cattle (Cappellozza et al., 2020; Colombo et al. 2020; Schubach et al., 2020), BW gain was not expected to differ herein given the short length of mBAS administration and the lack of major stressors during the final 7 days on feed. The mBAS replicates the composition of the original bovine appeasing pheromone that has calming effects when perceived by the animal, and shown to improve welfare and productivity of cattle exposed to stressful procedures (Cappellozza and Cooke, 2022). No treatment differences were observed (*P *= 0.73) for proportion of carcasses that graded Choice or Prime (Table 2), as mBAS was also not expected to affect marbling during the last week on feed.

**Table 2.** Body weight (**BW**) and carcass characteristics of steers assigned to pens that contained or not (**CON**; *n* = 6 pens, 477 steers) an oiler that delivered the maternal bovine appeasing substance (**mBAS**; *n* = 6 pens, 477 steers) during the last 7 d prior to slaughter in Exp. 1^1,2^

Item	CON	mBAS	SEM	*P*-value
Initial BW, kg	638	635	8	0.75
Final BW, kg	654	647	7	0.48
Average daily gain, kg	1.07	0.83	0.25	0.51
Hot carcass weight, kg	420	427	4	0.29
Carcass dressing, %	64.2	65.9	0.5	0.02
Carcasses classified as dark cutters, %	2.83	0.87	1.10	0.23
Carcasses graded Choice or Prime, %	81.1	78.5	5.2	0.73

^1^The oiler (Prairie Phoenix Cattle Care System; Whitehorse, SD) containing mBAS (Ferappease Finish Cattle 5%; FERA Diagnostics and Biologicals; College Station, TX) was designed to deliver 120 mL of mBAS per steer during a 7-d period. Oilers were checked daily and mBAS was replenished according to dosage when necessary.

^2^Initial BW was recorded when steers were assigned to experimental pens (mBAS or CON; 14.3 ± 2.0 d prior to slaughter), and final BW was recorded when steers were loaded for transport (48 km) to the packing plant (National Beef Packing Company; Liberal, KS). Carcass characteristics were reported by the packing plant, and carcass dressing was calculated according to final BW and hot carcass weight.

Carcass dressing was greater (*P *= 0.02) by 1.7-percentage points in mBAS steers (Table 2), which can be associated with reduced body tissue catabolism and less muscle glycogen depletion. One of the primary adrenocortical stress responses is to consume muscle glycogen stores and degrade hepatic, muscle, and adipose tissues to provide nutrients for homeostatic restoration (Nelson and Cox, 2005; Carroll and Forsberg, 2007). Muscle glycogen content directly impacts water holding capacity (**WHC**), as the glycogen molecule binds up to four times its weight in water (Olsson and Saltin, 1970). It seems plausible that mBAS administration increased carcass dressing by alleviating the stress elicited by the slaughter process, thus reducing body tissue wastage and increasing WHC of muscle cells. Nonetheless, treatment effects on carcass dressing were not sufficient to impact HCW (*P *= 0.29; Table 2).

Muscle glycogen is responsible for the formation of dark-cutting meat, as glycogen content is negatively associated with postmortem meat pH (Apple et al., 2005). No treatment differences were noted (*P *= 0.23) for proportion of carcasses classified as dark cutters herein (Table 2), although such outcome was decreased by 3-fold in mBAS compared with CON steers. The incidence of dark cutters in this experiment averaged 1.8% (17 dark cutters from 954 total carcasses), corroborating values reported by the 2016 National Beef Quality Audit (1.9%; Boykin et al., 2017). Dark cutting is a binary carcass response with low prevalence, and Wulf et al. (2002) reported that 1,150 experimental units would be required per treatment to detect a two-percentage point difference in this trait. Hence, additional research with greater statistical power is warranted to investigate the potential benefits of mBAS in mitigating the incidence of dark cutters.

### Experiment 2

No treatment effects were detected (*P *≥ 0.20) for BW parameters, as well as carcass marbling score, backfat thickness, LM area, yield grade, and proportion of carcasses that graded Choice or Prime (Table 3). As in Exp. 1, mBAS was not expected to affect BW gain, muscle development, or intramuscular and subcutaneous fat accretion during the final 7 d on feed. Carcass dressing was greater (*P *= 0.02) by 1.0-percentage point in mBAS steers (Table 3), but such difference was also insufficient to impact steer HCW (*P *= 0.47; Table 3). Steers from this experiment were transported for 720 km to the packing plant, and carcass dressing was calculated based on steer BW at loading and HCW. Hence, dressing calculation included the BW shrink caused by the 720-km transport, resulting in carcass dressing values below Exp. 1 and industry average (Davis et al., 2024). If a 4% pencil shrink is added to final BW to adjust for transport (González et al., 2012), the dressing percentage remains greater (*P *= 0.02) in mBAS compared with CON + (63.2 vs. 62.2%, respectively; SEM = 0.3).

**Table 3.** Body weight (**BW**) parameters, carcass characteristics, and plasma cortisol concentrations upon slaughter in steers assigned to pens that contained an oiler that delivered the maternal bovine appeasing substance (**mBAS**; *n* = 8 pens, 40 steers) or mineral oil (**CON**; *n* = 8 pens, 40 steers) during the last 7 d prior to slaughter in Exp. 2^1,2^

Item	CON	FERA	SEM	*P*-value
BW parameters				
Initial BW, kg	601	599	7	0.82
Final BW, kg	602	600	6	0.79
Average daily gain, kg	0.132	0.127	0.213	0.98
Carcass characteristics				
Hot carcass weight, kg	359	364	4	0.47
Carcass dressing, %	59.6	60.6	0.3	0.02
Marbling score	396	401	11	0.78
Backfat, cm	1.07	1.03	0.06	0.55
* Longissimus* muscle area, cm^2^	93.5	95.6	1.1	0.20
Yield grade	2.31	2.21	0.08	0.35
Carcasses classified as dark cutters, %	5.13	2.44	2.95	0.53
Carcasses graded Choice or Prime, %	46.1	51.2	7.9	0.65
Plasma cortisol upon slaughter, ng/mL	20.8	11.7	1.6	<0.01

^1^Oilers (Prairie Phoenix Cattle Care System; Whitehorse, SD) containing mBAS (Ferappease Finish Cattle 5%; FERA Diagnostics and Biologicals; College Station, TX) or mineral oil were designed to deliver 120 mL of mBAS per steer during a 7-d period. Oilers were checked daily and mBAS was replenished according to dosage when necessary.

^2^Steer initial BW was recorded 7 d prior to slaughter, and final BW was recorded when steers were loaded for transport (720 km) to the packing plant (Tyson; Amarillo, TX). Upon slaughter, a blood sample was collected during exsanguination into a blood collection tubes (Vacutainer, 10 mL; Becton Dickinson, Franklin Lakes, NJ) containing freeze-dried sodium heparin and hot carcass weight was recorded. Carcass dressing was calculated according to final BW and hot carcass weight. Trained personnel assessed carcass characteristics after a 24-h chill. Backfat thickness was measured at the 12th rib; marbling score: 300 = Slight^00^, 400 = Small^00^; yield grade calculated according to Lawrence et al. (2010).

Plasma concentration of cortisol upon slaughter was decreased (*P *< 0.01) by 44% in mBAS steers compared to CON+ (Table 3). This outcome supports our hypothesis and provides initial evidence that mBAS administration alleviated the stress elicited during processing for slaughter (Cappellozza and Cooke, 2022). Cortisol is paramount to the adrenocortical stress response (Sapolsky et al., 2000) and directly stimulates glycogen and muscle tissue breakdown (Nelson and Cox, 2005). Hence, the increase in carcass dressing noted herein and in Exp. 1 may be associated with less body tissue wastage and increased WHC of muscle cells in mBAS steers. Additional research is warranted to validate this rationale, including postmortem meat glycolytic potential, pH change, raw and cooked meat quality traits from cattle administered mBAS prior to slaughter (Wulf et al., 2002). No treatment effects were noted (*P *= 0.53) for proportion of carcasses classified as dark cutters, despite being decreased by 2-fold in mBAS compared with CON steers (Table 3). The incidence of dark cutters in this experiment (3.75%; 3 dark cutters from 80 carcasses) was above industry average (Boykin et al., 2017) and values from Exp. 1, which can also be associated with the long transport to the packing plant (Warren et al. 2010). Nonetheless, results from this experiment further suggest the potential benefits of mBAS in mitigating the incidence of dark-cutting carcasses.

### Overall conclusions

Administering mBAS to finishing cattle using oilers during the last 7 d on feed reduced the cortisol response elicited by the process of slaughter, which likely resulted in increased carcass dressing. The magnitude of such increase, however, was greater in Exp. 1 compared with Exp. 2. The CON pens in Exp. 1 did not contain oilers, which may have contributed as environmental enrichment to further mitigate stress in mBAS pens (Park et al., 2020). Nonetheless, mBAS and CON steers were exposed to stressful conditions after they were removed from the pens, which limits the potential impacts of oilers on results from Exp. 1. Other factors such as transport distance, small-pen vs. large-pen management, feedlot location, and packing plant procedures are more likely to have contributed to differences between experiments (Edwards-Callaway et al., 2020). Therefore, additional research is warranted to further characterize the benefits of mBAS administration to finishing cattle prior to slaughter, including mechanisms associated with postmortem glycogen depletion, muscle WHC, and dark-cutting carcasses.
